# Where Do Patients With Cirrhosis Die? A CDC WONDER Analysis From 1999 to 2020

**DOI:** 10.1002/jgh3.70205

**Published:** 2025-07-08

**Authors:** Areej Iftikhar, Fatima Noor Alam, Danish Ali Ashraf, Saad Hassan Qureshi, Muzamil Akhtar, Ayesha Islam Khan, Muhammad Raza, Raheel Qureshi, Raheel Ahmed

**Affiliations:** ^1^ Department of Medicine Allama Iqbal Medical College Lahore Pakistan; ^2^ Department of Medicine Services Institute of Medical Sciences Lahore Pakistan; ^3^ Department of Medicine Foundation University Medical College Islamabad Pakistan; ^4^ Department of Medicine Bahria University Health Sciences Karachi Pakistan; ^5^ Department of Medicine Gujranwala Medical College Gujranwala Pakistan; ^6^ Department of Medicine Jinnah Sindh Medical University Karachi Pakistan; ^7^ Department of Gastroenterology Queen Elizabeth Hospital Gateshead UK; ^8^ National Heart and Lung Institute Imperial College London UK

**Keywords:** disparities, liver cirrhosis, mortality trends, place of death

## Abstract

**Background:**

Liver Cirrhosis is a growing cause of morbidity and mortality worldwide, but significant knowledge gaps remain regarding disparities in the place of patient death. This study aims to analyze the place of death trends for cirrhosis patients in the United States from 1999 to 2020, to direct preventive and palliative care measures.

**Methods:**

In this descriptive study, death certificates were examined using the Centers for Disease Control and Prevention Wide‐Ranging Online Data for Epidemiologic Research (CDC WONDER) database, using ICD codes K70.3, K71.7, and K74. The variables studied were the year, sex, age, race, rural–urban, census regions, and state. Percentages of deaths in each location were trended over time for interpretation.

**Results:**

Between 1999 and 2020, a total of 1 090 420 deaths were attributed to cirrhosis. The majority of these fatalities occurred in inpatient medical facilities (48.75%), followed by the decedent's home (25.71%). Men consistently exhibited higher deaths in all settings, especially in medical facility—inpatient settings (31.39%), and the highest deaths were for the 55–64 age group. NH White population reported the highest mortality in all places of death, whereas the South led mortality records among census regions. The least deaths occurred in non‐core (non‐metro) areas, and state‐wise analysis revealed California to have the highest number of deaths (75 723), seconded by Texas (55 447).

**Conclusion:**

Mortality due to cirrhosis increased gradually from 1999 to 2019, with a steep rise observed in 2019–2020. Men, the White race, 55–65 years age bracket, and residents of rural and Southern census regions reported the highest number of deaths from cirrhosis.

## 1 Introduction

Cirrhosis is a prevalent cause of morbidity and mortality in people living with chronic liver disease (CLD) [1]. An estimated 1.5 billion people suffer from CLD around the globe, with a staggering 13% rise in global disease incidence since 2000 [2]. In the United States, end‐stage liver disease (ESLD) is claimed to be the 10th leading cause of mortality [3], despite multiple innovations in medical, radiological, and interventional approaches to patient diagnostic and palliative care. This increasing incidence of CLD is indeed correlated to a rise in cirrhosis cases, evidenced by various studies across different regions [4, 5]. CLDs such as Hepatitis C, alcoholic and nonalcoholic fatty liver disease, are seen to majorly increase the risk for the development of cirrhosis [5, 6], whereas autoimmune liver diseases, genetic pre‐dispositions, and prolonged exposure to certain medications are also believed to contribute a minor share [7].

In most cases however, a subject with cirrhosis remains clinically asymptomatic for a long time [8], exacerbating the risk of going undiagnosed and ultimately spiraling downwards to death. The progression from a compensated phase to a decompensated phase results in significant deterioration in state of health [6, 9], and complications like progressive portal hypertension, hepatic encephalopathy or hepatocellular carcinoma, further deteriorate patient prognosis [1, 2, 6, 7]. The prevention and management of complications as well as the risk‐stratification of patients requires well‐established contemporary estimates of ESLD epidemiology and place of patient death. In this regard, our study provides valuable insight into the interplay of personal, socioeconomic, and environmental factors that contribute to cirrhosis‐based death. This study is conducted thereof to suggest future directions and assist the healthcare system toward a more efficient, patient‐centered approach [9].

## 2 Methods

### 2.1 Study Setting and Population

This study adheres to the Strengthening the Reporting of Observational Studies in Epidemiology (STROBE) guidelines, focusing on analyzing the place of death trends in mortality due to cirrhosis in the United States from 1999 to 2020. The data was extracted from the Centers for Disease Control and Prevention Wide‐Ranging Online Data for Epidemiologic Research (CDC WONDER) database, which includes records of death certificates from all 50 states and the District of Columbia. Mortality data was identified using the International Classification of Diseases, Tenth Revision (ICD‐10) codes, that is, K70.3 (Alcoholic cirrhosis of liver), K71.7 (Toxic liver disease with fibrosis and cirrhosis of liver), and K74 (Fibrosis and Cirrhosis of liver), whereas other diseases of the liver (K76) were excluded. The Multiple Cause‐of‐Death certificates from public use records were studied to analyze deaths that were related to cirrhosis. On the death certificates, these deaths were defined as having cirrhosis stated either as a contributory or underlying cause of death. Institutional review board (IRB) approval was not required for this study, as we utilized a publicly available, de‐identified dataset provided by the government that does not involve any interaction with human subjects.

### 2.2 Data Abstraction

The extracted data included information on the location of death, year, total number of deaths, percent of total deaths, patient demographics, and US census regions. The places of death in our study were categorized as medical facility—inpatient, medial facility—outpatient/ER, decedent's home, hospice facility, and nursing home/long‐term care centers, whereas any places other than the aforementioned ones were labeled as “other.” We stratified our data based on three demographic variables namely, sex, age, and race/ethnicity. The categories used for segregating race and ethnicity included non‐Hispanic (NH) White, Black/African American, Hispanic/Latino, American Indian/Alaskan Native, and Asian/Pacific Islander. According to the US Census Bureau definitions, the census regions were classified as Northeast, Midwest, South, and West.

### 2.3 Statistical Analysis

The data were transferred to a Microsoft Excel sheet and the total number of deaths based on the four criteria was segregated according to the place of death. We estimated the annual incidence and age‐adjusted mortality rates (AAMRs) per 100 000 individuals from 1999 to 2020 by place of death and demographic variables, such as sex, race/ethnicity, state, and census, with 95% CIs to investigate the place of death in cirrhosis patients in the United States. The Joinpoint Regression Program (Joinpoint V 4.9.0.0, National Cancer Institute) was used to analyze the annual percent change (APC) with 95% CI in AAMR to measure the trends of place of mortality; however, the APCs and AAMRs do not apply to place of death analysis. Hence, the percentage of total deaths in each location was trended over time to see the overall picture. The Excel graphs were used for data interpretation and the aggregated data was displayed in a tabulated form (Table 1).

**TABLE 1:** Aggregated data for places of death by decedent's characteristics for cirrhosis (1999–2020).

Decedent's characteristics	Total *n* (%)	Medical facility—inpatient (%)	Medical facility—outpatient or ER (%)	Decedent's home (%)	Hospice facility (%)	Nursing home/long term care
Age, years						
35–44	68 966 (6.3)	39 030 (3.6)	4504 (0.4)	14 884 (1.4)	2970 (0.3)	2911 (0.3)
45–54	229 905 (21.1)	124 295 (11.4)	11 483 (1.1)	53 736 (4.9)	12 544 (1.2)	14 289 (1.3)
55–64	324 344 (29.7)	166 703 (15.3)	12 080 (1.1)	81 707 (7.5)	22 184 (2.0)	27 069 (2.5)
65–74	247 208 (22.7)	116 406 (10.7)	6679 (0.6)	67 747 (6.2)	16 884 (1.6)	30 119 (2.8)
75–84	156 871 (14.4)	62 058 (5.7)	3163 (0.3)	45 396 (4.2)	10 129 (0.9)	29 459 (2.7)
85+	49 793 (4.6)	15 314 (1.4)	817 (0.1)	14 193 (1.3)	3311 (0.3)	13 693 (1.3)
Sex						
Female	388 608 (35.6)	189 306 (17.4)	12 085 (1.1)	94 919 (8.7)	25 093 (2.3)	50 470 (4.6)
Male	701 812 (64.4)	342 309 (31.4)	27 693 (2.5)	185 458 (17.0)	43 557 (4.0)	67 395 (6.2)
Race						
American Indian or Alaska Native	20 901 (1.9)	11 249 (1.0)	959 (0.1)	4780 (0.4)	948 (0.1)	1837 (0.2)
Asian or Pacific Islander	22 662 (2.1)	12 928 (1.2)	958 (0.1)	5488 (0.5)	764 (0.1)	1669 (0.2)
Black or African American	106 023 (9.7)	61 592 (5.7)	5881 (0.5)	19 564 (1.8)	5159 (0.5)	8382 (0.8)
White	940 834 (86.3)	445 846 (40.9)	31 980 (2.9)	250 545 (23.0)	61 779 (5.7)	105 977 (9.7)
Hispanic Origin						
Hispanic or Latino	156 479 (14.4)	84 148 (7.7)	7185 (0.7)	39 126 (3.6)	7910 (0.7)	11 127 (1.0)
Not Hispanic or Latino	929 885 (85.3)	445 366 (40.8)	32 397 (3.0)	240 432 (22.1)	60 552 (5.6)	106 338 (9.8)
County classification						
Large metro	546 121 (50.1)	279 027 (25.6)	21 038 (1.9)	133 320 (12.2)	32 661 (0.7)	53 878 (4.9)
Medium/small metro	354 876 (32.6)	162 788 (14.9)	12 165 (1.1)	95 271 (8.7)	28 035 (2.5)	39 177 (3.6)
Rural	189 423 (17.4)	89 800 (8.2)	6575 (0.6)	51 789 (4.7)	7954 (0.7)	24 810 (2.3)
Census region						
Census region 1: Northeast	167 085 (15.3)	91 597 (8.4)	5800 (0.5)	35 784 (3.3)	7234 (0.7)	21 602 (2.0)
Census region 2: Midwest	209 787 (19.2)	99 580 (9.1)	7181 (0.7)	52 713 (4.8)	11 704 (1.1)	29 656 (2.7)
Census region 3: South	439 219 (40.3)	210 341 (19.3)	15 740 (1.4)	112 639 (10.3)	38 559 (3.5)	37 807 (3.5)
Census region 4: West	274 329 (25.2)	130 097 (11.9)	11 057 (1.0)	79 241 (7.3)	11 153 (1.0)	28 800 (2.6)
Total						
Number of deaths	1 090 420	531 615 (48.75)	39 778 (3.65)	280 377 (25.71)	68 650 (6.30)	117 865 (10.81)

## 3 Results

### 3.1 Overall Trends

**FIGURE 1:**
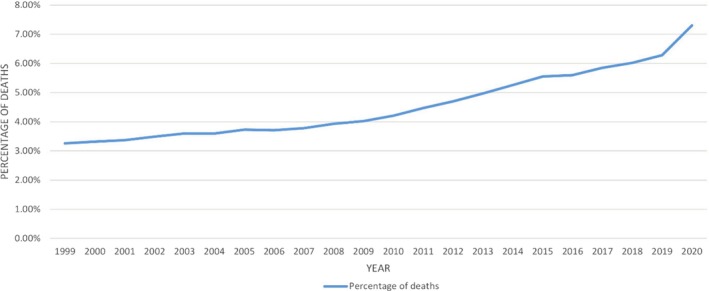
Overall mortality trend in the United States for cirrhosis from 1999 to 2020.

Between 1999 and 2020, 1 090 420 deaths (Male 64.36%; White 86.28%) were attributed to cirrhosis. Yearly deaths in cirrhosis increased by 124.14% over the study period, with 35 528 deaths in 1999 to 79 631 in 2020 (Figure 1).

**FIGURE 2:**
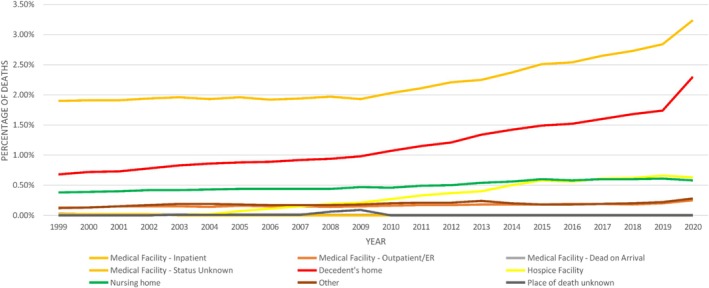
Mortality trends in places of death resulting from cirrhosis stratified by years.

The inpatient medical facility remained the most common location of death throughout the study (48.75%), followed by the decedent's home (25.71%) and nursing home/long‐term care (10.81%). The proportion of deaths in medical facilities decreased—inpatient (58.5%) and ER (4%) in 1999 to 44% and 3% in 2020, respectively. By contrast, the proportion of deaths increased from 21% at Home in 1999 to 32% in 2020 (Figure 2; Figure S1) (Table 1).

## 4 Demographic Trends

### 4.1 Sex

**FIGURE 3:**
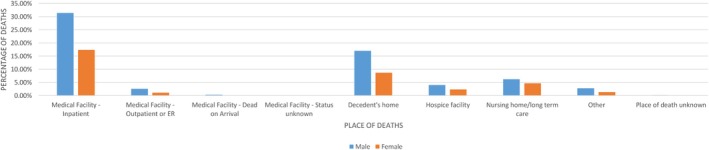
Mortality trends in places of death resulting from cirrhosis stratified by gender.

The largest percentage of individuals who died of Cirrhosis were males. Most of the fatalities took place in medical facility—inpatient for both genders—male (31.39%) and female (17.36%). The second highest proportion of deaths in both genders; male (17.01%) and female (8.70%) was observed at the decedent's home. The least number of deaths were recorded in medical facility—status unknown; male (0.06%) and female (0.03%) (Figure 3).

### 4.2 Age Groups

**FIGURE 4:**
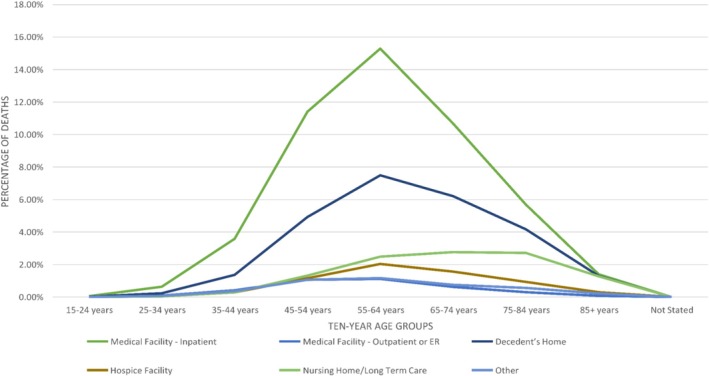
Mortality trends in places of death resulting from cirrhosis stratified by age groups.

For our 10‐year age group analysis, the highest proportion of deaths was seen in individuals aged 55–64 years (15.29%) in medical facility—inpatient. Moreover, there was a similar trend in deaths seen in individuals of the 10‐year age group 55–64 years in medical facility—outpatient or ER (1.11%), decedent's home (7.49%), hospice facility (2.03%) and others (1.16%). On the contrary, a greater proportion of deaths in nursing home/long‐term care was among the patients aged 65–74 years (2.76%). The lowest number of deaths occurred in the younger population (15–24 years) for all the places of death. However, a decline in trend was seen from the age group 55–64 years to 85+ years for most of the places of death (Figure 4).

### 4.3 Race and Ethnicity

**FIGURE 5:**
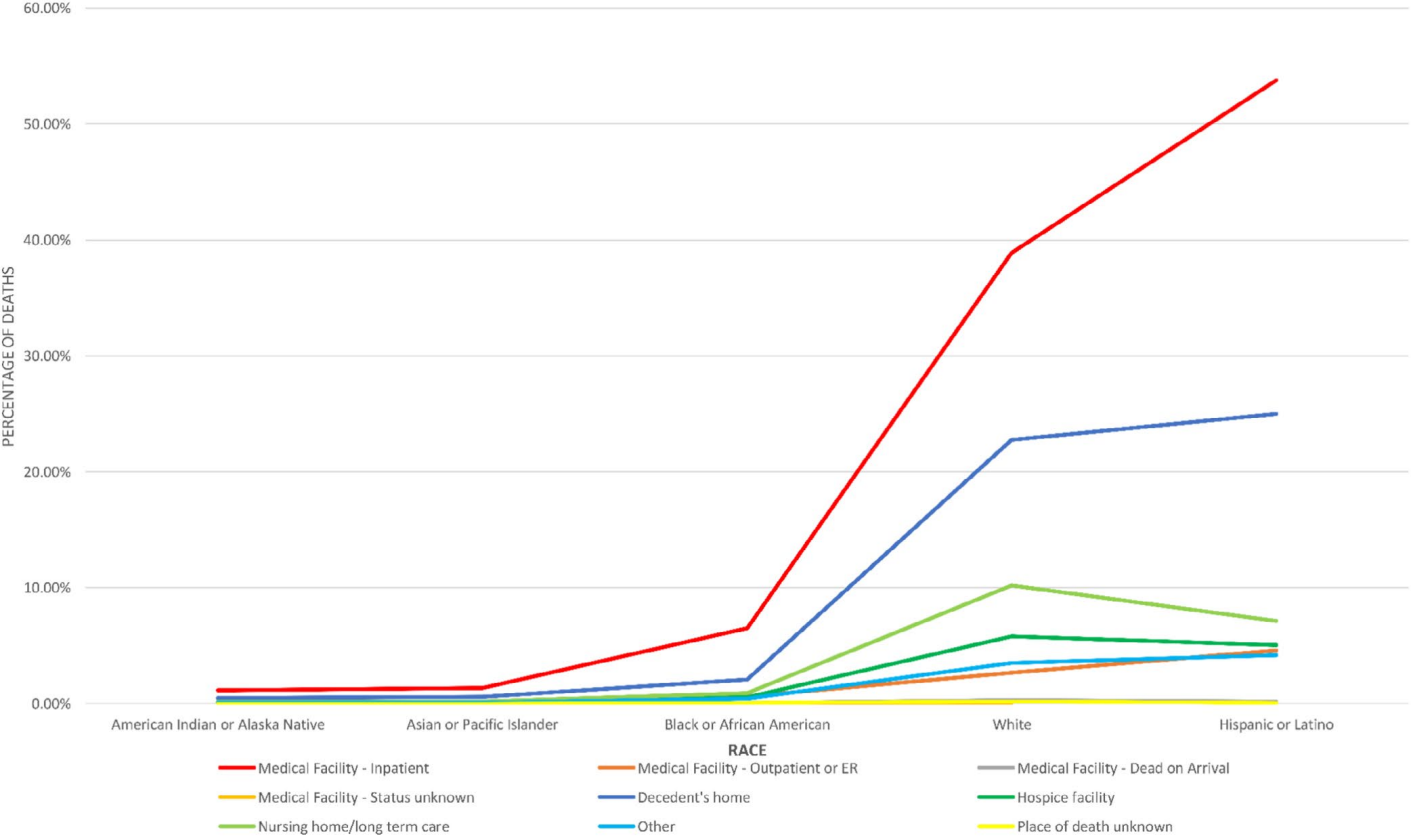
Mortality trends in places of death resulting from cirrhosis stratified by race.

Throughout the studied years, the highest numbers of deaths were seen in the White population in all locations (hospital 35.89%, home 25.06%, hospice 6.39%, and nursing facilities 6.41%) as compared to other races. The numbers were found to be 445 846 in the inpatient settings, 31 980 for the outpatient settings, 250 545 for the decedent's home, and 61 779 for the hospice facility settings. However, even in the White population, a greater proportion of deaths was observed in home and hospice facility settings over the years rather than in hospital and nursing facilities. On the other hand, the smallest number of deaths was reported for Alaskan natives in all locations of death in our study with the numbers being 11 249 inpatient deaths, 959 outpatient deaths, 4780 at decedent's home, and 948 hospice facility deaths. The proportion of deaths of Alaskan Natives, Asians, Blacks, and Hispanics at all places remained nearly constant, however among these populations, higher death was seen in hospital and home settings as compared to hospice and nursing facilities (Figure 5).

## 5 Geographical Patterns

### 5.1 Urban–Rural

**FIGURE 6:**
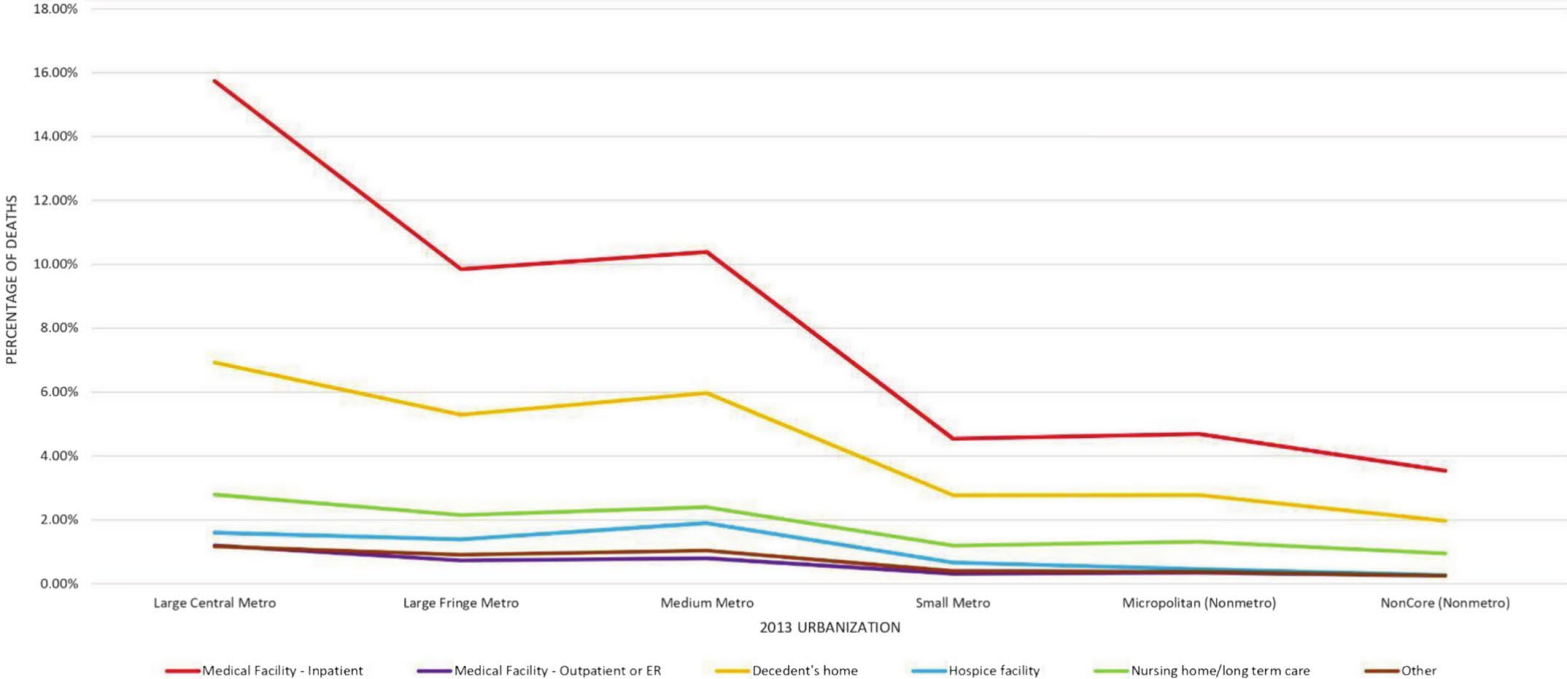
Mortality trends in places of death resulting from cirrhosis stratified by county classification.

Throughout the study period, the largest number of deaths was recorded in Large Central Metro (17.7% in medical facilities, 6.93% in decedent's Home, 2.79% in nursing home, 1.6% in hospice facility, 1.17% in others, and 0.04% in unknown places), followed by medium metro (11.26% in Medical Facilities, 5.97% in decedent's Home, 2.4% in nursing home, 1.9% in hospice facility, 1.04% in Others and 0.03% in unknown places). The least number of deaths was recorded in non‐core (non‐metro) (3.83% in medical facilities, 1.97% in decedent's home, 0.95% in nursing home, 0.27% in hospice facility, 0.25% in others and 0.02% in unknown places) (Figure 6).

### 5.2 Census Regions and State‐Wise

**FIGURE 7:**
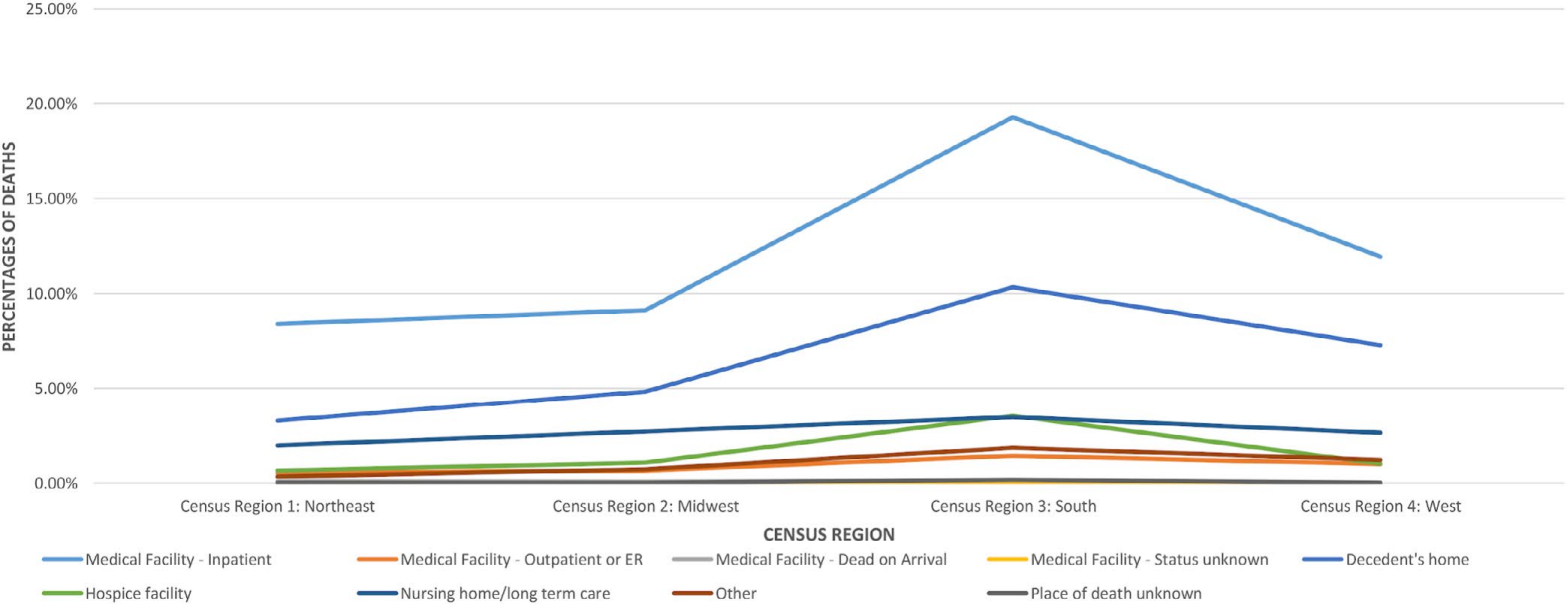
Mortality trends in places of death resulting from cirrhosis stratified by census regions.

Compared to the other parts of the country, the greatest number of deaths was seen in the South. Among the deaths in the South, the leading place of death was medical facility—inpatient (19.29%) and the least deaths were reported in medical facility—status unknown (0.04%). The minimum proportion of deaths occurred in the Northeast region for most of the places of death. California was reported to have the highest death number (75 723), Texas ranking second (55 447), and Florida third (33 002). The lowest number of deaths was found in Wyoming (877) (Figures 7 and S2–S4).

## 6 Discussion

Between 1999 and 2010, cirrhosis‐related mortality in the United States exhibited a concerning upward trend, reversing prior declines. This increase is largely attributed to a surge in alcohol‐associated liver disease (ALD), particularly among younger adults [10, 11]. Literature suggests individuals aged 25–34 experienced the highest annual rise in cirrhosis mortality due to alcohol abuse, averaging 10.5% per year from 2009 to 2016 [10]. Additionally, the rising prevalence of nonalcoholic fatty liver disease (NAFLD) driven by escalating obesity rates, a delayed access to healthcare services in some regions, and a rising incidence of Hepatitis C infection further contributed to the rising burden [11]. This trend may have been exacerbated by the 2008 financial crisis in the United States, which correlated with increased alcohol misuse among young adults due to economic stressors [12].

As the prevalence of cirrhosis in the United States rose across these past two decades [13, 14], the disparity in palliative and quality‐of‐life (QOL) care in cirrhosis‐affected individuals across different populations has become more significant and demands attention. This study, henceforth, analyzes the disparities in cirrhosis‐related mortality trends in the United States for the years 1999–2020.

The place of death observed with the highest cirrhosis mortality was the inpatient facility (*n* = 531 615), seconded by the decedent's home (*n* = 280 377). Death rates were significantly higher among the White race (*n* = 783 942) and the age group of 55–65 years. Male patients were seen to have a twofold tendency compared with females to die at their homes and/or inpatient facilities. Rural areas and Southern census regions were revealed to be predominantly affected, showing higher proportional mortality rates than geographical counterparts.

Inpatient facilities being the most common place of death for cirrhosis emphasize that a majority of cirrhosis patients need to undergo critical inpatient interventions to prevent health deterioration and death. Decompensated cirrhosis is a frequent reason for admission to medical emergencies, typically leading to a prolonged hospital stay [15]. A high symptom burden and a deteriorated QOL [16], with insubstantial treatment options and an undetermined prognosis [17], serve as a nidus for high inpatient mortality.

The decedent's home was the second major place of death across all age groups and races. As place is integral to a patient's palliative care and impacts proximity to family, available resources, and patient comfort [18], most patients prefer home to be their last resort. Factors like the minimization of medical interventions for a patient at home, the provision of a personalized comfortable space, and more patient control over surroundings are significant reasons why patients with morbidity would prefer an in‐home death in contrast to a Nursing/Long term care center. Prior literature evidences many patients dying in institutions have unmet needs for symptom amelioration, communication, emotional care, and respect [19]. It is however of note that the preferred place of death for a patient may change with progression to severe disease, or if the necessary support to remain at home is lacking [20] which includes formal caregivers for both emotional and physical support of terminally ill patients.

Although the trends for in‐home deaths have increased consistently for the past 2 decades, a steep rise was observed after 2019 (1.74% in 2019 to 2.3% in 2020). This finding is due largely to the COVID‐19 Pandemic that altered end‐of‐life (EOL) quality and care for patients across all disease spectra. This global pandemic ranks as the deadliest disaster in American history [21], which diverted resources away from the management of multiple chronic diseases, including cirrhosis [22]. Key findings in a research on early mortality for acute cirrhosis decompensation deduced a higher disease severity during COVID‐19 when compared to pre‐COVID‐19, with a significant rise in readmission rates and mortality in this period [23], underscoring its fatal influence.

Hospice and nursing home facilities demonstrated low mortality rates, which could be attributed mainly to limited knowledge and subsequently limited utilization of such facilities by patients [24, 25]. Given the rapidly growing population of older adults in minority populations, many of whom will face advanced morbidities like cirrhosis or ESRD, the lack of awareness regarding the utility and efficacy of such palliative facilities needs due attention and intervention.

Gender stratification demonstrated that almost double the males (31.39%) died of cirrhosis in inpatient facilities compared to females (17.36%) and similar trends were seen for decedents' homes (17.01% males, 8.70% females). Literature establishes significant gender disparities in bile acid metabolism, gene signaling, and gut microbiota that might contribute to a higher prevalence of fatty liver disease and hepatocellular carcinoma in men [26]. Men with diagnosed cirrhosis who die of a subsequent cardiac morbidity like heart failure are also seen to be significantly higher compared to women with established cirrhosis [27]. Of relevance is a contemporary study on the outcomes of liver transplants (LT) in Polycystic Liver Disease (PLD) patients that demonstrated women had a substantially lower risk of mortality post‐transplantation than men [28]. This reflects that women not only have a lower risk of developing morbid liver dysfunction but also have a protective effect against fibrosis [29] and mortality after palliative and curative interventions. A detailed review of the causative factors of such gender‐specific disparities, however, lies beyond the scope of this research and duly requires further study.

Based on 10‐year age groups, the greatest number of deaths was found in the 55 to 64‐year age group for both inpatient and hospice deaths (166 703 and 22 184 deaths respectively). Nursing home/long‐term care facility deaths among older age groups (aged 55 and above) were notably higher than younger patient groups, highlighting the disparities in EOL management of older patients. It is established that the “aged liver” is more sensitive to acute and chronic injury and is at greater risk of severe fibrosis or cirrhosis [30]. Cirrhosis may be underdiagnosed in older people, likely due to the presence of fewer clinical signs at presentation and less frequent use of invasive diagnostic modalities [31]. A contemporary research suggests older patients are more likely to have a “comfort therapy only” code in hospitals compared to young patients [32] and are more likely to be discharged from the hospital to home, nursing or hospice facilities, circumventing futile in‐patient measures, thus contributing to higher mortality in other places of death.

A greater proportion of NH Whites (40.89%) and NH African Americans (5.65%) died in inpatient facilities compared to NH Alaskan Natives (1.03%) or NH Asians (1.19%). Given that minority populations have less access to quality care, less insurance coverage for placement in hospice facilities, and limited affordability of high‐quality resources, a greater mortality in these settings when compared to socioeconomically privileged regions is justifiable [33]. Banerjee et al. [34] argue that an important element of institutional and individual bias exists in access to quality education and healthcare, which strongly influences the EOL care of minority individuals. A study conducted by VoPham T. showed that in comparison to non‐Hispanic Whites, Hispanic patients had a greater risk for HCC (hepatocellular carcinoma) overall as well as with cirrhosis etiology, whereas non‐Hispanic Blacks had a relatively lower risk [35, 36]. Moreover, Asian patients had higher HCC rates caused by HC/HBV‐cirrhosis, demonstrating that race and ethnicity is a principal predictor for developing hepatic decompensation and mortality [35, 36]. Disparities also exist in receiving critical life‐saving interventions like LD [37], accentuating the racial bias. An integrative approach to patients irrespective of race and ethnicity, and education to both patients and healthcare workers about the benefits of palliative care and hospice, may help bridge these gaps in the future.

Significant disparities in cirrhosis mortality exist across US census regions and the rural–urban gradient, attributable to an interplay of sociodemographic, economic, and healthcare access factors [14, 38, 39]. Higher risk factors like alcoholism, cigarette smoking, obesity, and physical inactivity, coupled with significantly higher poverty, unemployment, and illiteracy rates in rural subgroups [40, 41, 42], are strong predictors of higher mortality. Literature suggests that for patients with ESLD, greater travel distances to liver transplant centers and liver specialists contribute to higher mortality rates, highlighting how living in marginalized suburban areas impacts disease prognosis and hinders effective EOL care for patients [43] Similarly, stratification by census regions showed the highest death rates for the Southern region in all places of death, with inpatient deaths (210 341 deaths) significantly higher than decedent's home (112 639) or hospice facility settings (38 559), consistent with prior studies [44]. A higher concentration of racial and ethnic minorities in the Southern Census, who experience higher cirrhosis mortality rates due to systemic healthcare inequities, fewer healthcare facilities and specialists' availability, lead to delayed diagnosis and poorer management. Differences in healthcare delivery and accessibility across the regions, that is, differences in hospital costs and outcomes for cirrhosis complications, further widen the disparity [9, 45].

These patterns point to critical gaps in early detection, outpatient management, and coordinated care pathways across the United States.

Targeted public health interventions on high‐risk groups to improve socioeconomic deprivation, health awareness and specialized healthcare access across the country are the need of the hour. To address this, public health policies must prioritize preventive strategies such as routine liver function screening in high‐risk populations, early referral systems, and education on modifiable risk factors like alcohol consumption and viral hepatitis. Strengthening community‐based care and improving transitions from inpatient to outpatient settings could reduce avoidable deaths. The integration of these insights into national liver disease strategies are required, to curb the growing burden of cirrhosis and improve patient outcomes across all levels of care.

## 7 Limitations

Our research is to be reviewed in the context of a few limitations. First, although the CDC WONDER database allows us to retrospectively study disease outcomes in a large population, it is deficient in comprehensive baseline disease characteristics of patients based on symptoms, disease progression, or imaging studies. Second, no information is provided relevant to the patient's access to treatment modalities, therapies received, or related aspects which could have further navigated in interpreting the disparities in the location of patient death. Thirdly, mortality trends and disparities in place of patient death that were observed in the United States may not apply elsewhere, considering a wide range of factors that influence a patient's perception of their preferred place of death. At last, a limitation that pertains to the database itself is that the precise cause of death may be open to varying interpretations in some cases, ultimately depending upon the judgment of the provider submitting the death certificate on the CDC WONDER website.

## 8 Conclusion

In conclusion, this study reveals the greatest proportion of cirrhosis‐related deaths in the years 1999 to 2020 occurred in inpatient settings, with White males, aged 55–64 years, and residents of the Southern Census region and rural areas being more likely to die of cirrhosis. No significant decrease was observed in mortality trends of cirrhosis patients in any place of death for the past 20 years, which reflects a limited implementation of any successful interventional strategies among these patients. Ensuring that health funding and policy‐making needs of the US population are met through cost‐effective and evidence‐based interventions is crucial to direct a better prognosis, especially in socio‐economically deprived subgroups. As palliative and hospice care programs and measures can ameliorate the burden of symptoms, reduce hospitalization, and improve EOL care for such patients, efforts to improve these facilities and educate vulnerable populations on relevant aspects would prove favorable in the future.

## Ethics Statement

The authors have nothing to report.

## Conflicts of Interest

The authors declare no conflicts of interest.

## Supporting information


**Figure S1.** Overall trend for age‐adjusted mortality rate (per 100 000) in the United States for cirrhosis from 1999 to 2020.
**Figure S2.** Mortality trends in places of death resulting from cirrhosis stratified by states.
**Figure S3.** Age‐adjusted mortality rates of cirrhosis stratified by state per 100 000.
**Figure S4.** Visual depiction of state‐wise mortality resulting from cirrhosis from 1999 to 2020.

## Data Availability

The data utilized in this research were obtained from the CDC WONDER (Wide‐Ranging Online Data for Epidemiologic Research) database.
