# Supplementary material for: Where Do Patients With Cirrhosis Die? A CDC WONDER Analysis From 1999 to 2020
Source: JGH Open. 2025 Jul 8;9(7):e70205. doi: 10.1002/jgh3.70205 (PMC12238664; doi:10.1002/jgh3.70205)
Supplement: Supplementary file 1 — Figure S1. Overall trend for age‐adjusted mortality rate (per 100 000) in the United States for cirrhosis from 1999 to 2020. Figure S2. Mortality trends in places of death resulting from cirrhosis stratified by states. Figure S3. Age‐adjusted mortality rates of cirrhosis stratified by state per 100 000. Figure S4. Visual depiction of state‐wise mortality resulting from cirrhosis from 1999 to 2020. [file JGH3-9-e70205-s001.docx]

**Supplementary Files**


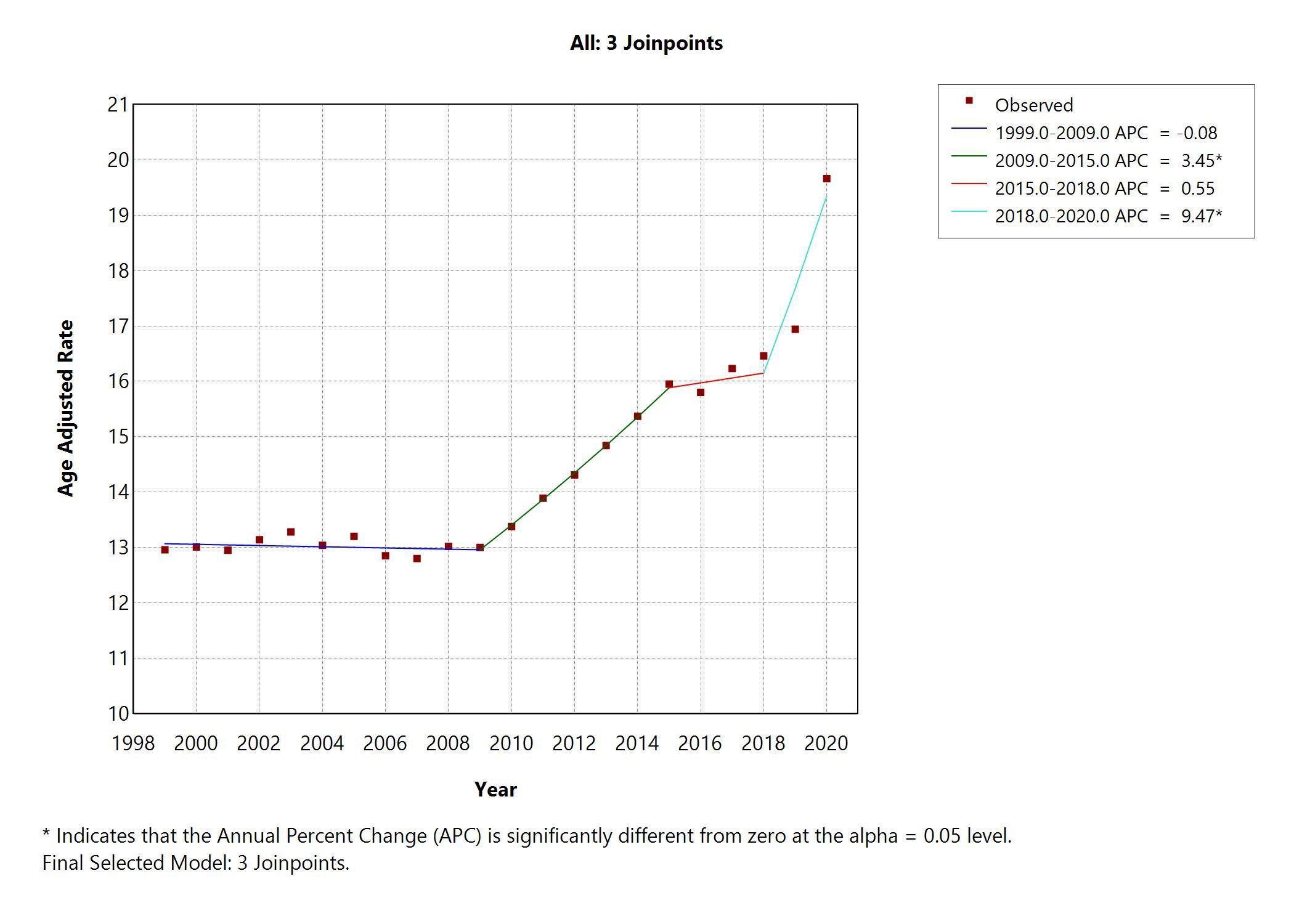


***Fig.1*** *Overall trend for age adjusted mortality rate (per 100,000) in U.S. for cirrhosis from 1999 to 2020*


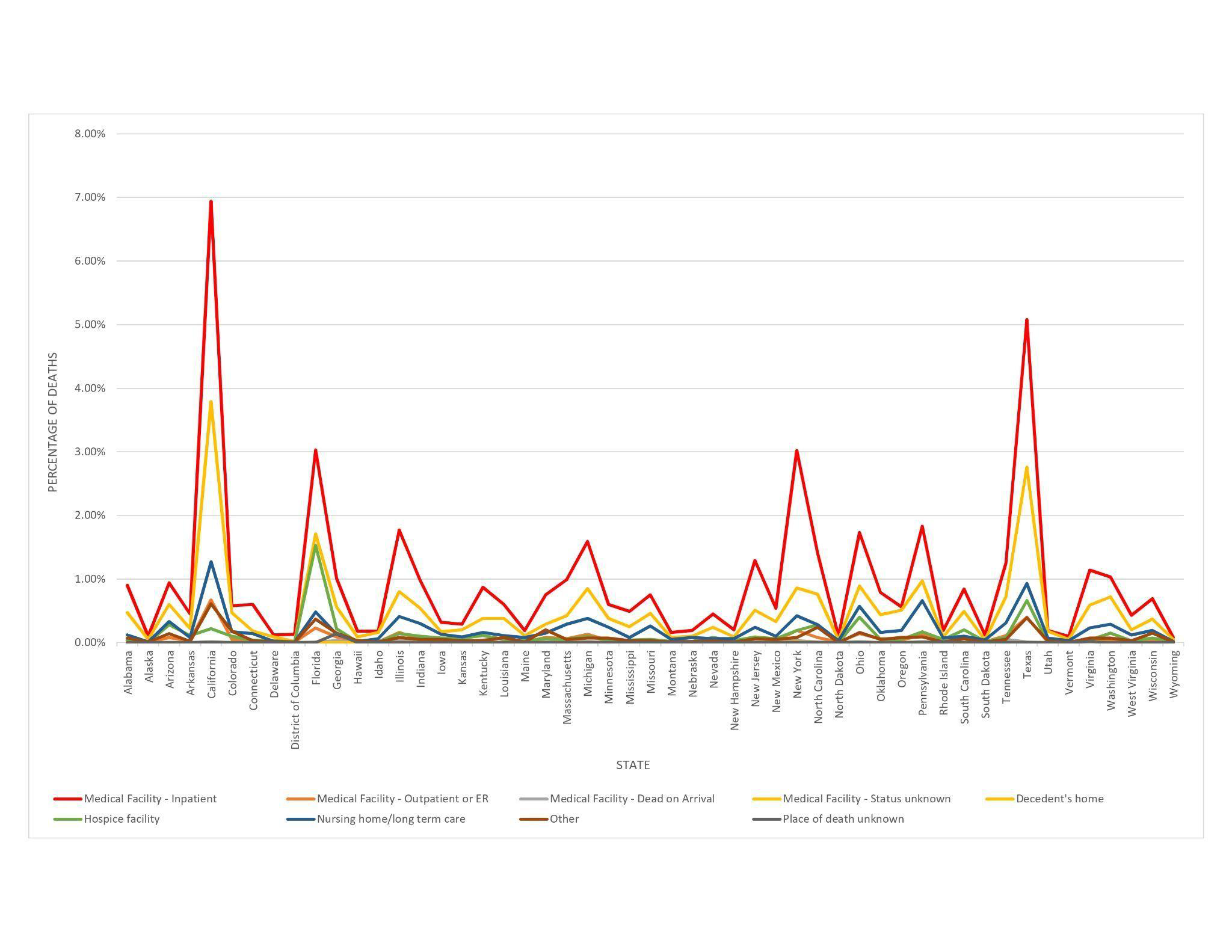


***Fig.2*** *Mortality trends in places of death resulting from cirrhosis stratified by states*


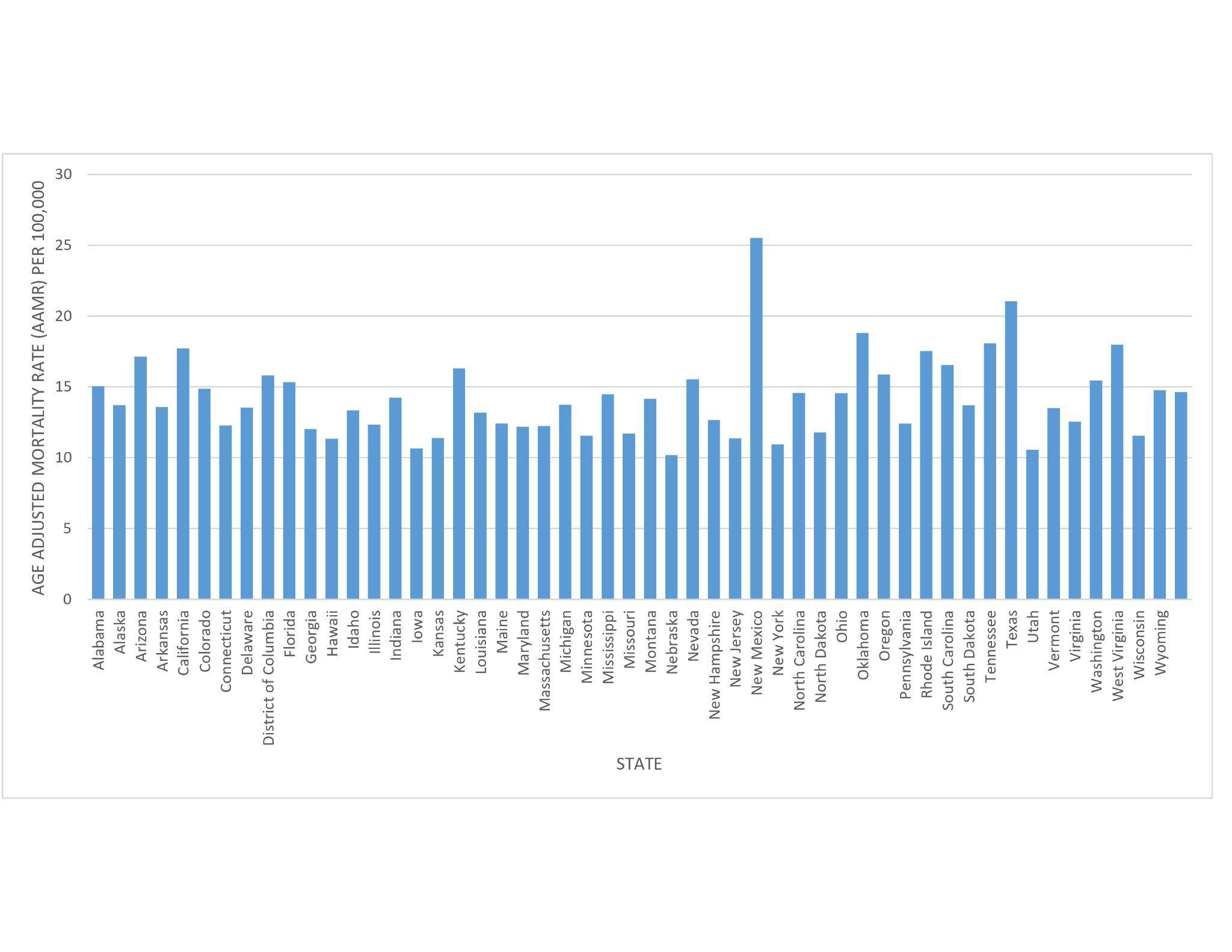


***Fig.3*** *Age adjusted mortality rates of cirrhosis stratified by state per 100,000*


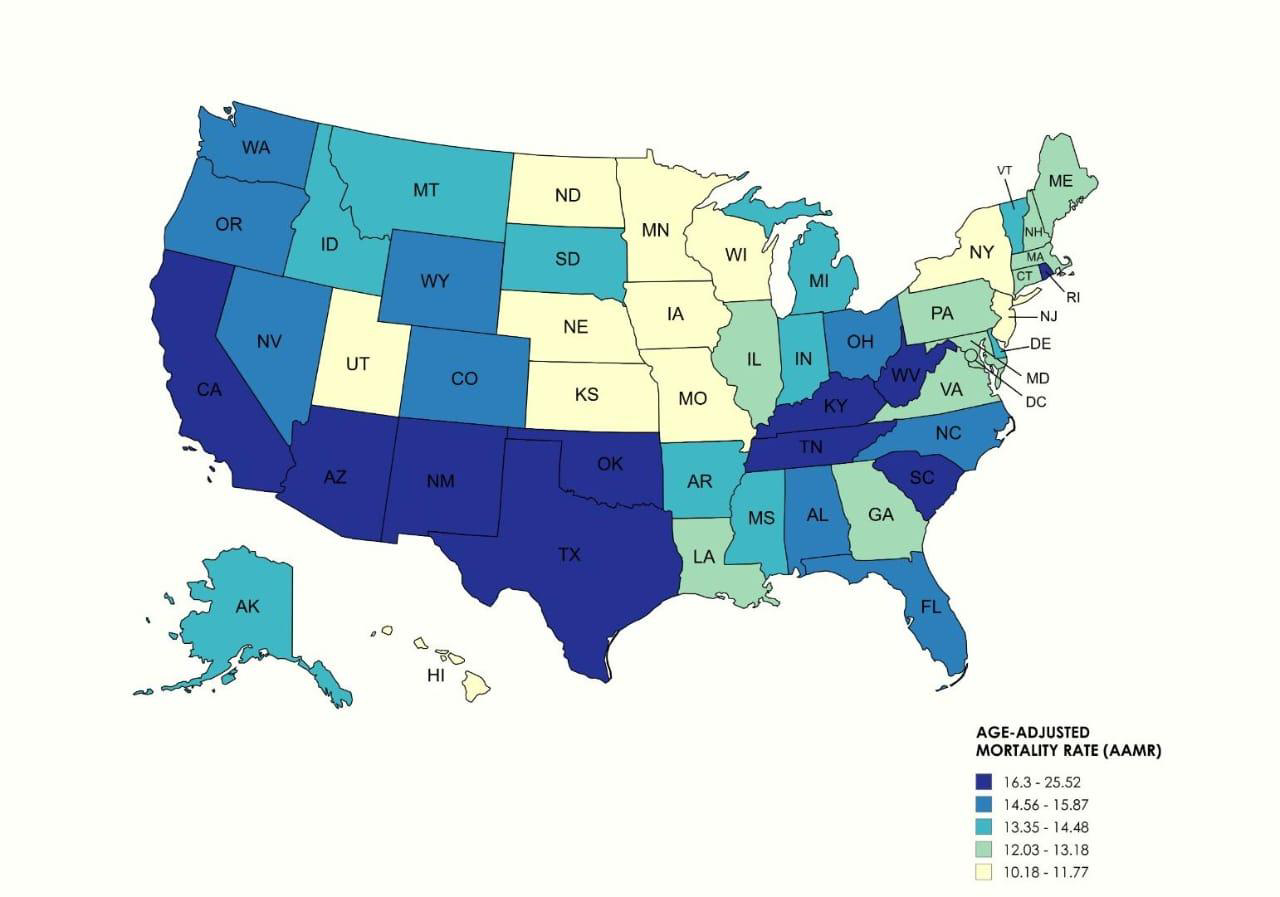


***Fig.4*** *Visual depiction of state-wise mortality resulting from cirrhosis from 1999 to 2020*
